# Factors associated with health behaviors in preventing non-communicable diseases among older adults living alone in poverty in Japan

**DOI:** 10.3389/fpubh.2023.1207334

**Published:** 2023-09-08

**Authors:** Yuki Imamatsu, Etsuko Tadaka

**Affiliations:** ^1^Department of Community Health Nursing, Graduate School of Medicine, Yokohama City University, Yokohama City, Japan; ^2^Department of Community and Public Health Nursing, Graduate School of Health Sciences and Faculty of Medicine, Hokkaido University, Sapporo, Japan

**Keywords:** older adults living alone in poverty, health behavior, prevention of non-communicable diseases, influencing factors, community commitment

## Abstract

**Introduction:**

Older adults who live alone in poverty are highly susceptible to non-communicable diseases and other adverse conditions owing to health disparities resulting from social structures. However, the factors associated with health behavior to prevent non-communicable diseases in this population are rarely explored. The purpose of this study was to identify factors associated with health behavior to prevent non-communicable diseases among older adults living alone in poverty.

**Methods:**

We conducted a self-administered mail survey covering 2,818 older adults living alone who were receiving public assistance, randomly selected from lists of individuals receiving national public assistance in all 1,250 local social welfare offices across Japan. A total of 1,608 individuals completed the questionnaire, a valid response rate of 57.1%. Respondents’ mean age was 74.5 years (standard deviation = 6.7), and 52.9% were women. The study variables included demographic characteristics, scores on a health behavior scale for older adults living alone and receiving public assistance (HBSO), and individual and community-related factors.

**Results:**

Logistic regression analysis revealed that the individual factor of having a health check-up in the past 12 months [odds ratio (OR): 1.45, 95% confidence interval (CI): 1.10–1.91] and the community-related factors Lubben social network scale score (OR 1.15, 95% CI: 1.12–1.18) and Community Commitment Scale score (OR: 1.04, 95% CI: 1.00–1.08) were significantly associated with HBSO scores.

**Conclusion:**

To improve health behavior among older adults living alone in poverty in Japan, social structures, such as lowering mental barriers to the detection, treatment, and management of non-communicable diseases and developing human resources, should be changed to provide social support, such that these individuals are not only dependent on family and friends.

## Introduction

A University of Oxford study on social determinants of health reported that economic disparities are associated with health disparities, and that international interest in health disparities has increased over the last two decades (1). Populations at lower socioeconomic levels generally experience poorer health and have higher rates of disability and mortality (2–5). In addition, recent studies have shown that populations at lower socioeconomic levels are more likely to have undesirable habits, such as smoking, that may affect disease prevention (6). The association between economic and health disparities is now considered less from a macro level, as a problem of disparities between high-income countries (HIC) and low- and middle-income countries (LMIC), and more from a micro level, as health disparities between different regions within the same country (and even within the same region) related to differences in personal income. Therefore, the issue of increasing health disparities is a serious and growing challenge in HIC, including Japan, and is not limited to LMIC. Non-communicable diseases (NCDs) are a major contributor to health disparities. They account for a large proportion of chronic illnesses and constitute one of the most important challenges in reducing health disparities. The World Health Organization (WHO) (7) has identified the main NCDs as cardiovascular diseases, cancers, chronic respiratory diseases, and diabetes, and has suggested that NCDs may be caused by inappropriate health behavior. The WHO (7) has also reported that NCDs kill 41 million people each year, accounting for 71% of all deaths worldwide. Socioeconomic factors are strongly related to the occurrence of NCDs. People who are more socioeconomically disadvantaged are more likely to develop NCDs and to become seriously ill (7). Thus, people with low social status or those living in poverty require support because they are more susceptible to NCDs and related health problems. Furthermore, older adults with low socioeconomic status are a particularly important risk group for NCDs (7, 8). Among them, older adults living alone are recognized as being vulnerable to NCDs and in need of additional support. Previous studies have reported that older adults living alone are more likely than other household members to fail to undergo medical check-ups and to be more likely to skip meals, and not go out (9–11). These risks are further increased by poverty. Therefore, older adults living alone in poverty (OAPs), who are more susceptible to diseases such as NCDs and other harmful aspects of health disparities owing to social structures, are a main target for intervention. It is also important to examine those factors related to social structures that may need to be changed to protect this population.

There are 36.4 million older adults in Japan, comprising 29.1% of the population. Japan has the highest worldwide proportion of elderly people as a percentage of the total population, and is becoming a hyper-aged society at an unparalleled rate (12). The second and fifth leading causes of death in Japan are cardiac and cerebrovascular diseases, respectively, and NCDs are the leading cause of death overall (13). Furthermore, NCDs contribute to the requirement of long-term care, which hinders the length of healthy life expectancy (14). In Japan, differences in healthy life expectancy between prefectures are up to 2.7 years (15), and underlying differences in socioeconomic status may explain these disparities (16). Therefore, the prevention of NCDs is important to extend healthy life expectancy and address health disparities in Japan. Any examination of socioeconomic disparities and poverty in Japan must also consider the role of the social security system (“Seikatsu-hogo” in Japanese), which provides public assistance. The Japanese Constitution guarantees a minimum standard of living for individuals who have difficulty establishing a sound economic basis using their own resources and abilities, a measure designed to support people living in poverty. According to the Japanese Ministry of Health, Labour and Welfare (17), approximately 1.64 million households receive public assistance; of the total number of households, 55.3% (the largest proportion) comprise older adults. Furthermore, more than 90% of older adult households (approximately 830,000 households) consist of individuals that live alone. Additionally, more than 90% of older adult households that receive public assistance also receive medical assistance (18). Thus, older adults who live alone comprise the largest proportion of public assistance recipients, and they represent a vulnerable population that often requires medical assistance and experiences high risk in both aspects of daily living and chronic illness. Thus, OAPs are an important target group when addressing health disparities in Japan.

Health behavior is a key concept in the prevention of NCDs, which are considered preventable with appropriate health behavior (19). Gochman (20) defined health behavior as not only observable actions, behavioral patterns, and habits but also personal attributes related to health maintenance and wellness, restoration, and health improvement. Previous studies have reported that desirable health behavior among older adults can lower the risk of heart failure (21), prevent the development of diabetes (22), and lower the risk of cancer death (23). Thus, health behavior clearly has a positive impact on preventing NCDs during the aging process, and improving appropriate health behaviors is important for the prevention of these diseases in older adults. However, older adults with low socioeconomic status, including OAPs, are reported to have difficulty performing health behaviors that are generally considered desirable (24, 25).

Therefore, we developed the Health Behavior Scale for Older Adults Living Alone Receiving Public Assistance (HBSO). This is a validated instrument that assesses health behaviors associated with the prevention of NCDs in OAPs (26). The scale assesses a range of health behaviors, and thus permits an examination of health behavior factors associated with the prevention of NCDs in this population. The clinical and policy significance of this study is that it used a self-administered questionnaire survey to clarify OAP health behaviors potentially associated with NCDs. This approach allowed us to examine social structures that may help to reduce health disparities while respecting aspects related to the self-perception of personal power and practical skills for daily health in OAPs.

Thus, the purpose of this study was to identify factors associated with health behaviors in preventing NCDs among OPAs in Japan.

## Materials and methods

### Participants

In 2020, we conducted a self-administered mail survey covering 2,818 OAPs who were randomly selected from lists of individuals receiving public assistance (Seikatsu-hogo) in all 1,250 local social welfare offices across Japan. We conducted the survey with the cooperation of social welfare offices to obtain a necessary sample size of OAPs. The researchers had no direct access to OAPs, for privacy protection reasons. We sent a letter to all social welfare offices explaining the purpose of the study and requesting their cooperation. In total, 155 offices (12.5%) agreed to participate in the survey. Staff from the 155 offices then randomly distributed questionnaires to a total of 2,818 OAPs. In the questionnaire, participants were asked to reflect on their behavior and to answer all questions themselves. We allowed welfare office staff to complete the survey on behalf of study participants only if participants requested this by themselves. As a result, 1,280 (45.4%) participants responded to the survey, among which 1,069 (37.9%) provided valid responses suitable for analysis. The criteria for valid responses were age ≥ 65 years, no missing responses to any HBSO items, and no missing responses regarding health check-up status.

### Measurements

#### Demographic characteristics

We collected the following demographic characteristics of OAPs: age, sex, and certification of long-term care needs (Support need levels 1 and 2, and Care need levels 1 to 5; a higher number indicates a greater need) under the Long-Term Care Insurance system of Japan, which is a part of the social security system.

#### Dependent variable

The “Health behavior scale for older adults living alone receiving public assistance” (HBSO) is a valid instrument for assessing health behavior for the prevention of NCDs in OAPs (26). An internal characteristic in OAPs is a sense of lacking personal power that manifests as a decrease in positive feelings (e.g., low self-esteem, self-usefulness, and self-affirmation); this characteristic leads to poor health and is shaped by socioeconomic background (27–30). Being in a socioeconomically difficult situation exposes individuals to everyday stresses, as well as discrimination and prejudice (31). These experiences rob OAPs of the perception of having power because they create negative feelings (31, 32). This discourages individuals from making healthy choices, which in turn makes it difficult to engage in appropriate health behaviors (31, 33–35). Therefore, Factor I of the HBSO comprises items that measure internal characteristics among OAPs and assesses “self-perception of personal power.” Additionally, the characteristics of external factors that affect OAPs include a lack of skills necessary to properly engage in health behavior, as well as knowledge and experience (36, 37); this results from a lack of opportunities or access to opportunities to acquire this knowledge and experience (37, 38). Factor II of the HBSO comprises items that measure external characteristics of OAPs and “assesses practical skills for daily health.” The strength of this subjective scale is that it is a self-administered questionnaire that can reveal the social structures that promote or inhibit health behaviors among OAPs.

The HBSO has a Cronbach’s alpha of 0.75 in Japan, which conveys the internal consistency of the scale, and includes 10 items, with five in each of the subscales “Self-perception of personal power” and “Practical skills for daily health.” Each item is scored on a four-point Likert-type scale as follows: 0 = Disagree, 1 = Disagree to a certain extent, 2: Agree to a certain extent, and 3: Agree. A higher overall score (range 0–30) indicates a higher degree of engaging in health behavior to prevent NCDs. No cutoff point for the HBSO has been determined; thus, we set a cutoff point based on the distribution of our study population.

### Independent variables

#### Individual factors

In this study, individual factors included availability of a family doctor, status of a health check-up in the past year, smoking habits, and alcohol consumption habits. Availability of a family doctor and health check-up status were selected as an objective health indicator. Responses to the question “Do you have a family doctor?” and “Have you had a regular health check-up in the last year?” were scored as follows: “Yes” = 1 point and “No” = 0 points. Smoking habits and alcohol drinking were assessed using participants’ responses to the question “Are you in the habit of smoking/drinking alcohol?” and were scored as follows: “Yes” = 1 point and “No” = 0 points.

#### Community/environmental factors

We assumed that community/environment factors could be represented by the participants’ social networks and sense of community. Social networks were measured using the Japanese version of the Lubben Social Network Scale (LSNS-6) (39). This scale has six items that measure the size, closeness, and frequency of contact with the respondent’s social network of family and friends, scored using a six-point Likert scale. Scores range from 0 to 30, with higher scores indicating a greater social network of family and friends. Sense of community was measured using the Community Commitment Scale (CCS) (40). This scale has a Cronbach’s alpha of 0.75 among local volunteers and consists of eight items, four in each of the subscales of socializing and belonging. Responses were measured using a four-point Likert-type scale where 0 = not confident at all, 1 = slightly unconfident, 2 = slightly confident, and 3 = fully confident. A higher overall score (range 0–24) indicates a higher degree of community commitment.

### Statistical analysis

The groups with high and low HBSO scores were analyzed separately. Then, on the basis of previous studies that conducted stratified analysis, differences between HBSO scores and the independent variables were compared using *t*-tests and χ^2^ tests [43]. Logistic regression analysis was performed to identify the key determinants of HBSO scores. Binomial logistic regression analysis was carried out for factors that differed between the two groups, to account for collinearity. The results were considered statistically significant with *p* < 0.05 a 95% confidence interval (CI) that did not include 1. IBM SPSS software version 28.0 (IBM Corp., Armonk, NY, United States) was used for the analysis.

### Ethical considerations

All participants were informed, both in writing and verbally, of the study purpose and methods and were assured that refusal to participate in the study or subsequent withdrawal would not result in any negative implications. Moreover, we explained that participation was voluntary and that completing and returning the questionnaire indicated their consent to participate in the study. This research was conducted in accordance with the 1964 Declaration of Helsinki and its amendments as well as the ethical guidelines for life sciences and medical research involving human subjects of the Ministry of Health, Labour and Welfare of Japan. The study was approved by the institutional ethical review board of the School of Medicine, Yokohama City University (approval no. A200700005).

## Results

### Dependent variable

Table 1 shows the HBSO scores. We set a cutoff of 16/17 in our analysis. Of the 1,069 participants, 546 (51.1%) were in the high-score group and 523 (48.9%) were in the low-score group. The mean ± standard deviation (SD) and range of HBSO scores was 16.5 ± 6.1 (0–30) for all participants, 21.3 ± 3.3 (17–30) for the high-score group, and 11.5 ± 3.6 (0–16) for the low-score group. Additionally, the overall score for “Self-perception of personal power” (Factor I) on the HBSO was 7.5 (SD = 3.8) and that for “Practical skills for daily health” (Factor II) was 9.0 (SD = 3.5) (Table 1).

**Table 1:** Univariate analysis of the HBSO.

	All(*n* = 1,069)	High(*n* = 546)	Low(*n* = 523)
Mean(SD)	16.5(6.1)	21.3(3.3)	11.5(3.6)
FactorI	7.5(3.8)		
FactorII	9.0(3.5)		
Median	17.0	21.0	12.0
Mode	15	17	16
Range	0–30	17–30	0–16
Skewness	−0.175	−0.716	0.964
Kurtosis	−0.448	0.445	0.315

### Demographic characteristics

Table 2 shows the demographic characteristics of the included participants. Respondents’ mean overall age was 74.5 years (SD = 6.7 years); that of the high-score group was 75.0 years (SD = 6.7 years), and that of the low-score group was 74.0 years (SD = 6.6 years). The proportion of women in the total population was 52.9%, that in the high-score group was 63.4%, and that in the low-score group was 41.9%. The duration of receiving public assistance was 9.0 years (SD = 6.5 years) in the group overall, 8.9 years (SD = 6.1 years) in the high-score group, and 9.2 years (SD = 6.9 years) in the low-score group (Table 2).

**Table 2:** Test for difference between two groups of related factors of the HBSO.

	Overall(*n* = 1,069)		High(*n* = 546)		Low(*n* = 523)		*p*-value
	*n* or mean	% or SD	*n* or mean	% or SD	n or mean	% or SD	
Demographic characteristics							
Age	74.5	6.7	75.0	6.7	74.0	6.6	0.071
Sex							
Female	565	52.9	346	63.4	219	41.9	<0.001
Missing	13	1.0	5	0.9	8	1.5	
Certification for Long-term care need							
No/Independence	709	11.6	366	67.0	343	65.6	0.017
Support need levels 1–2	128	37.9	66	12.1	62	11.9	
Care need levels 1–2	64	20.0	25	4.6	39	7.5	
Care need levels 3–5	23	5.9	5	0.9	18	3.4	
Missing	145	13.6	84	15.4	61	11.7	
History of receiving public assistance	9.0	6.5	8.9	6.1	9.2	6.9	0.323
Individual factors							
Availability of a family doctor							
existence	899	84.1	480	87.9	419	80.1	0.002
Missing	12	1.1	6	1	6	1.1	
Health check-ups in the last 12 months							
receive	542	50.7	316	57.9	226	43.2	<0.001
Smoking habit							
None	440	41.2	264	48.4	176	33.7	<0.001
Missing	26	2.4	17	3.1	9	1.7	
Alcohol drinking habit							
None	773	72.3	418	76.6	355	67.9	0.005
Missing	46	4.3	22	4.0	24	4.6	
Community/Environment factors							
LSNS-6	8.2	6.0	10.6	6.2	5.7	4.8	<0.001
CCS	11.1	3.9	11.7	3.4	10.5	4.2	<0.001

### Independent variables

Table 3 lists factors related to HBSO scores in the univariate analysis (independent variables). The demographic characteristics with significant differences with respect to HBSO scores were sex (*p* < 0.001) and certification of a long-term care need (*p* = 0.017). Individual factors related to significant differences in the HBSO scores were availability of a family doctor (*p* = 0.002), health check-up in the past 12 months (p < 0.001), smoking (p < 0.001), and alcohol drinking (*p* = 0.005). Significant community/environmental factors related to HBSO scores were LSNS-6 score (p < 0.001) and CCS score (p < 0.001). Thus, after considering multicollinearity, six of the eight factors were selected as independent variables and were included in the final multivariate logistic regression analysis (Table 2).

**Table 3:** Logistic analysis of related factors of the HBSO.

	*p*-value		OR	(95% Cl)	
Age	0.085		1.018	0.998	1.039
Sex	0.559		0.996	0.984	1.009
Health check-ups in the last 12 months	0.004	***	1.484	1.134	1.942
Smoking habit	0.522		1.000	0.999	1.001
LSNS-6	<0.000	***	1.152	1.122	1.183
CCS	0.012	*	1.048	1.011	1.088

### Factors related to HBSO scores

Table 3 shows the related factors following multiple logistic analysis of HBSO scores. Factors that were associated with HBSO scores were health check-up in the past 12 months (odds ratio [OR]: 1.45, 95% CI: 1.10–1.91), LSNS-6 score (OR: 1.15, 95% CI: 1.12–1.18), and CCS score (OR: 1.04, 95% CI: 1.00–1.08) (Table 3).

## Discussion

To our knowledge, this was the first study to examine health behaviors and related factors among OAPs in Japan. The clinical and policy implications of this study were elucidated in terms of health behaviors among OAPs and related factors, assessed using a self-administered questionnaire survey. This approach allowed us to examine social structures that can help to reduce health disparities while respecting aspects of “self-perception of personal power” and “practical skills for daily health” among OAPs. The participants in this study were drawn from a national sample of OAPs in Japan. A comparison between our study participants and demographic data from a national survey on public assistance (18) revealed the following. Our participants were similar in age composition, with a mean age of 74.5 years in the current study, compared with 75.5 years in the previous survey. The percentage of women was 52.9% in the current study, compared with 52.1% in the previous survey. Comparing the results of the National Survey of Public Assistance with the results of the present survey, we consider that our sample was representative of the population, given the similarity of basic attributes such as average age and sex ratio. The relevant factors in this study included both individual and community/environmental factors, with individual factors considered to indicate health-related actions taken by individual OAPs.

In this study, the individual factor found to be significantly associated with HBSO scores was having a health check-up in the past 12 months. According to previous studies, individuals who do not undergo medical checkups (41) tend to be men and unmarried. Additionally, previous studies (42) have shown that the factors that contribute to older adults not receiving health checkups are not physical factors, such as aging, declining physical function, or experience of falls but rather the influence of mental factors, such as self-assessment of health and subjective sense of well-being. This may be the reason for lower scores for HBSO Factor I, “Self-perception of personal power,” than for Factor II, “Practical skills for daily health” in this study and the higher number of undiagnosed OAPs in the group with low HBSO scores. However, OAPs in Japan may have mental barriers to the detection, treatment, and management of NCDs. This result is because the current system in Japan is based on the assumption that people with higher educational levels, occupational status, and better-quality lifestyle, on average, will use the system. Thus, a social structure that lowers mental barrier to the detection, treatment, and management of NCDs in this population may need to be considered.

Among significant community-related factors for OAPs in our study, the average LSNS-6 score was 8.2 (SD = 6.0) in the overall group, 10.6 (SD = 6.2) in the group with high HBSO scores, and 5.7 (SD = 4.8) in the group with low HBSO scores. Previous studies have found average LSNS-6 scores of 15.9 (SD = 4.65) for the age group 65–74 years and 15.6 (SD = 5.66) for the age group 75 years and above (39). The average LSNS-6 score of older adult caregivers caring for a spouse or child was 13.4 (SD = 5.9) in one study (43). Moreover, our results showed that the average CCS score among OAPs overall was 11.1 (SD = 3.9), with 11.7 (SD = 3.4) in the group with high HBSO scores and 10.5 (SD = 4.3) in the group with low HBSO scores. Previous studies found an average CCS score of 14.5 (SD = 4.1) for the general urban older population (44) and 17.2 (SD = 3.7) for older people participating in community activities in urban areas (45). Similar to results of the LSNS-6, OAPs in our study were found to have lower CCS scores than older adults who were actively engaged in community activities, as well as older adults in general and even physically frail older adults. When considering community/environmental factors together with HBSO score, OAPs struggle with acquiring practical skills for daily health and have poor self-perception of personal power because they have very few social networks and a poor sense of community, lacking a sense of belonging and socialization within the community. A social structure that creates a social environment where OAPs can feel a sense of belonging and socialization, as well as development of social support personnel in the community, such that OAPs are not only dependent on family and friends, needs to be considered.

In conclusion, according to our findings regarding individual and community/environmental factors associated with HBSO scores among OAPs, the following three social structural changes are necessary to effectively enhance health behavior in this population: creation of a social environment to promote a sense of belonging and socialization among OAPs, as well as finding a place in the community; development of social support personnel in the community such that OAPs are not only dependent on family and friends; and lowering mental barriers among OAPs to the detection, treatment, and management of NCDs.

### Limitations

This study had several limitations. First, the cross-sectional design meant that determining causal relationships between HBSO scores and factors related to the individual or the community/environment was not possible. Therefore, longitudinal and interventional studies are needed to assess health behaviors in OAPs and relationships within their community and social network, as well as to examine how these change over time. Second, this study only included 12.5% of all municipalities in Japan, which might have biased the results. The fact that the study was conducted during the coronavirus-19 pandemic, which reduced the opportunities for face-to-face contact, may have made it more difficult for both the research field offices and the study participants to take part in the study. In addition, because we targeted OAPs in Japan’s community care system, which provides public assistance for health check-ups and medical visits, caution is needed when adapting this approach for use with populations served by other community care systems. Future studies should include municipalities with OAP populations that did not participate in this study and other HIC countries to ensure that the findings are more representative. Third, community and environmental factors were measured only in terms of sense of community and social networks among OAPs. However, community- or environment-related factors that enhance health behaviors to prevent NCDs in OAPs may also depend on other factors, such as the local physical environment and support from local government social welfare and medical personnel. Therefore, local environmental factors related to the health behavior of OAPs need to be further examined.

## Data availability statement

The raw data supporting the conclusions of this article will be made available by the authors, without undue reservation.

## Ethics statement

The studies involving human participants were reviewed and approved by institutional ethical review board of the School of Medicine, Yokohama City University. The patients/participants provided their written informed consent to participate in this study.

## Author contributions

YI and ET contributed to developing the concept, designing the study, and interpreting, drafting, and revising the manuscript. YI was responsible for data analysis. ET was responsible for data collection and responsible for acquiring Institutional Review Board (IRB) approval for this study, study supervision, and reporting of the results. All authors contributed to the article and approved the submitted version.

## Conflict of interest

The authors declare that the research was conducted in the absence of any commercial or financial relationships that could be construed as a potential conflict of interest.

## Publisher’s note

All claims expressed in this article are solely those of the authors and do not necessarily represent those of their affiliated organizations, or those of the publisher, the editors and the reviewers. Any product that may be evaluated in this article, or claim that may be made by its manufacturer, is not guaranteed or endorsed by the publisher.
